# Cognitive effects of unilateral thalamotomy for tremor: a meta-analysis

**DOI:** 10.1093/braincomms/fcac287

**Published:** 2022-11-04

**Authors:** Camryn R Rohringer, Isabella J Sewell, Shikha Gandhi, Jonah Isen, Benjamin Davidson, Melissa McSweeney, Walter Swardfager, Nadia Scantlebury, Richard H Swartz, Clement Hamani, Peter Giacobbe, Sean M Nestor, Yana Yunusova, Benjamin Lam, Michael L Schwartz, Nir Lipsman, Agessandro Abrahao, Jennifer S Rabin

**Affiliations:** Hurvitz Brain Sciences Program, Sunnybrook Research Institute, Toronto, ON M4N 3M5, Canada; Hurvitz Brain Sciences Program, Sunnybrook Research Institute, Toronto, ON M4N 3M5, Canada; Hurvitz Brain Sciences Program, Sunnybrook Research Institute, Toronto, ON M4N 3M5, Canada; Hurvitz Brain Sciences Program, Sunnybrook Research Institute, Toronto, ON M4N 3M5, Canada; Hurvitz Brain Sciences Program, Sunnybrook Research Institute, Toronto, ON M4N 3M5, Canada; Division of Neurosurgery, Department of Medicine, Sunnybrook Health Sciences Centre, University of Toronto, Toronto, ON M4N 3M5, Canada; Harquail Centre for Neuromodulation, Sunnybrook Research Institute, Toronto, ON M4N 3M5, Canada; Hurvitz Brain Sciences Program, Sunnybrook Research Institute, Toronto, ON M4N 3M5, Canada; Hurvitz Brain Sciences Program, Sunnybrook Research Institute, Toronto, ON M4N 3M5, Canada; Harquail Centre for Neuromodulation, Sunnybrook Research Institute, Toronto, ON M4N 3M5, Canada; Hurvitz Brain Sciences Program, Sunnybrook Research Institute, Toronto, ON M4N 3M5, Canada; Division of Neurology, Department of Medicine, Sunnybrook Health Sciences Centre, University of Toronto, Toronto, ON M4N 3M5, Canada; Hurvitz Brain Sciences Program, Sunnybrook Research Institute, Toronto, ON M4N 3M5, Canada; Division of Neurosurgery, Department of Medicine, Sunnybrook Health Sciences Centre, University of Toronto, Toronto, ON M4N 3M5, Canada; Harquail Centre for Neuromodulation, Sunnybrook Research Institute, Toronto, ON M4N 3M5, Canada; Hurvitz Brain Sciences Program, Sunnybrook Research Institute, Toronto, ON M4N 3M5, Canada; Harquail Centre for Neuromodulation, Sunnybrook Research Institute, Toronto, ON M4N 3M5, Canada; Department of Psychiatry, Sunnybrook Health Sciences Centre, University of Toronto, Toronto, ON M4N 3M5, Canada; Hurvitz Brain Sciences Program, Sunnybrook Research Institute, Toronto, ON M4N 3M5, Canada; Harquail Centre for Neuromodulation, Sunnybrook Research Institute, Toronto, ON M4N 3M5, Canada; Department of Psychiatry, Sunnybrook Health Sciences Centre, University of Toronto, Toronto, ON M4N 3M5, Canada; Hurvitz Brain Sciences Program, Sunnybrook Research Institute, Toronto, ON M4N 3M5, Canada; Rehabilitation Sciences Institute, University of Toronto, Toronto, ON M5G 1V7, Canada; Department of Speech-Language Pathology, University of Toronto, Toronto, ON M5G 1V7, Canada; KITE, Toronto Rehabilitation Institute, University Health Network, Toronto, ON M5G 2A2, Canada; Hurvitz Brain Sciences Program, Sunnybrook Research Institute, Toronto, ON M4N 3M5, Canada; Division of Neurology, Department of Medicine, Sunnybrook Health Sciences Centre, University of Toronto, Toronto, ON M4N 3M5, Canada; Hurvitz Brain Sciences Program, Sunnybrook Research Institute, Toronto, ON M4N 3M5, Canada; Division of Neurosurgery, Department of Medicine, Sunnybrook Health Sciences Centre, University of Toronto, Toronto, ON M4N 3M5, Canada; Hurvitz Brain Sciences Program, Sunnybrook Research Institute, Toronto, ON M4N 3M5, Canada; Division of Neurosurgery, Department of Medicine, Sunnybrook Health Sciences Centre, University of Toronto, Toronto, ON M4N 3M5, Canada; Harquail Centre for Neuromodulation, Sunnybrook Research Institute, Toronto, ON M4N 3M5, Canada; Hurvitz Brain Sciences Program, Sunnybrook Research Institute, Toronto, ON M4N 3M5, Canada; Harquail Centre for Neuromodulation, Sunnybrook Research Institute, Toronto, ON M4N 3M5, Canada; Division of Neurology, Department of Medicine, Sunnybrook Health Sciences Centre, University of Toronto, Toronto, ON M4N 3M5, Canada; Hurvitz Brain Sciences Program, Sunnybrook Research Institute, Toronto, ON M4N 3M5, Canada; Harquail Centre for Neuromodulation, Sunnybrook Research Institute, Toronto, ON M4N 3M5, Canada; Division of Neurology, Department of Medicine, Sunnybrook Health Sciences Centre, University of Toronto, Toronto, ON M4N 3M5, Canada; Rehabilitation Sciences Institute, University of Toronto, Toronto, ON M5G 1V7, Canada

**Keywords:** thalamotomy, cognition, ventral intermediate nucleus (Vim) of the thalamus, verbal fluency, tremor

## Abstract

Tremor is a debilitating symptom that can lead to functional impairment. Pharmacotherapy is often successful, but up to 50% of patients are resistant to medications or cannot tolerate side effects. Thalamotomy to the ventral intermediate nucleus of the thalamus is a surgical intervention for refractory tremor. Thalamotomy surgeries include radiofrequency and incisionless procedures, such as Gamma Knife radiosurgery and magnetic resonance-guided focused ultrasound. Cognitive changes following thalamotomy have been inconsistently reported across studies. We performed a meta-analysis to summarize the impact of unilateral thalamotomy to the ventral intermediate nucleus of the thalamus across multiple cognitive domains. We searched MEDLINE, Embase Classic, Embase and EBM Reviews for relevant studies. Neuropsychological tests were categorized into seven cognitive domains: global cognition, verbal memory, non-verbal memory, executive function, phonemic fluency, semantic fluency and visuospatial processing. We calculated standardized mean differences as Hedges’ g and 95% confidence intervals of the change between pre- and postoperative cognitive scores. Pooling of standardized mean differences across studies was performed using random-effects models. Risk of bias across studies and quality of evidence for each cognitive domain were assessed with the National Institute of Health quality assessment tool and the GRADEpro Guideline Development Tool, respectively. Of the 1251 records reviewed, eight studies met inclusion criteria. We included 193 patients with essential tremor, Parkinson’s disease, or multiple sclerosis in the meta-analysis. There was a small significant decline in phonemic fluency [standardized mean difference = −0.29, 95% confidence interval: (−0.52, −0.05), *P* = 0.017] and a trend towards a decline in semantic fluency [standardized mean difference = −0.19, 95% confidence interval: (−0.40, 0.01), *P* = 0.056]. No postoperative changes were observed in the other cognitive domains (*P* values >0.14). In secondary analyses, we restricted the analyses to studies using magnetic resonance-guided focused ultrasound given its growing popularity and more precise targeting. In those analyses, there was no evidence of cognitive decline across any domain (*P* values >0.37). In terms of risk of bias, five studies were rated as ‘good’ and three studies were rated as ‘fair’. According to GRADEpro guidelines, the certainty of the effect for all cognitive domains was low. This study provides evidence that unilateral thalamotomy to the ventral intermediate nucleus of the thalamus is relatively safe from a cognitive standpoint, however, there may be a small decline in verbal fluency. Magnetic resonance-guided focused ultrasound might have a more favourable postoperative cognitive profile compared with other thalamotomy techniques.

## Teaser

Based on a meta-analysis of eight studies, Rohringer, Sewell *et al.* report that unilateral ventral intermediate nucleus thalamotomy for tremor is relatively safe from a cognitive standpoint. The only cognitive domain to show a small postoperative decline was verbal fluency, all other domains remained stable.

## Graphical Abstract

**Graphical Abstract:**
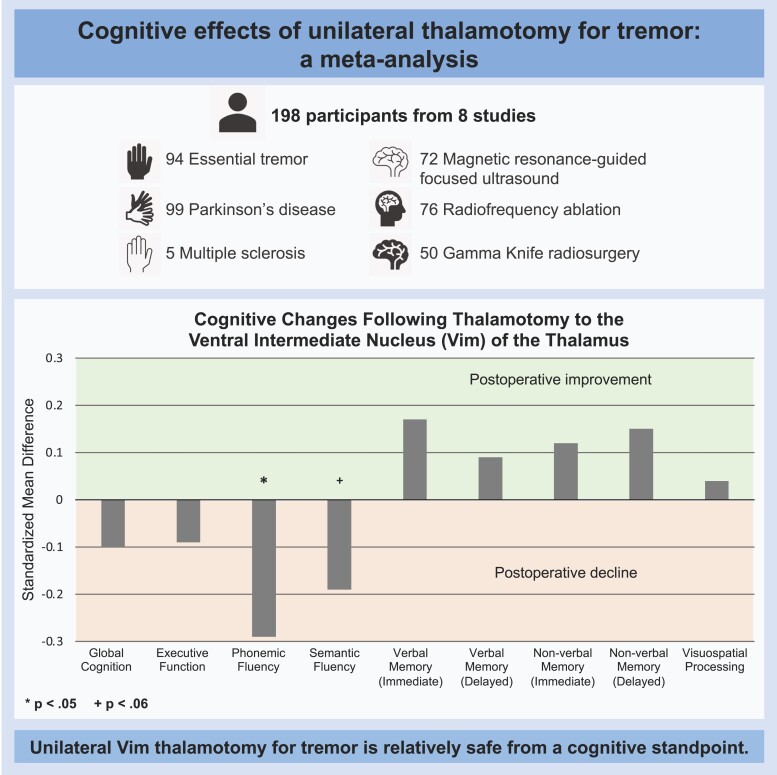

## Introduction

Tremor is a debilitating symptom observed across multiple neurological disorders, including essential tremor, Parkinson’s disease and multiple sclerosis. Severe tremor can interfere with activities of daily living, such as drinking, eating, dressing and writing, leading to a reduced quality of life.^1^ The first line of treatment for tremor is pharmacological,^2^ however, up to 50% of patients are resistant to medications or cannot tolerate side effects.^3,4^ In these cases, surgical interventions targeting the ventral intermediate nucleus (Vim) of the thalamus may be considered.^5^

Vim thalamotomy is an effective treatment for patients with refractory tremor.^5^ Thalamotomy can be performed with various techniques, including radiofrequency, Gamma Knife radiosurgery and magnetic resonance-guided focused ultrasound (MRgFUS). Radiofrequency is an established thalamotomy technique that involves placing an electrode through the brain parenchyma to ablate the Vim.^6^ It is an open surgical procedure and is associated with risks, such as intracerebral haemorrhage and infection.^7,8^ More recently, incisionless thalamotomy procedures have been developed, such as Gamma Knife radiosurgery and MRgFUS.^9,10^ These interventions involve focusing multiple beams of radiation (Gamma Knife) or ultrasound waves (MRgFUS) on brain targets without the need to open the skull.^5,6^ A limitation of Gamma Knife radiosurgery is that tremor improvements are delayed by weeks or months, which can result in lesions that are larger than intended.^11^ By contrast, the effects of MRgFUS are immediate, allowing real-time monitoring of the lesion size based on patients’ clinical response and feedback.^12^ These advantageous features have contributed to the growing popularity of MRgFUS thalamotomy for tremor.^13^

While Vim thalamotomy procedures effectively mitigate tremor,^14^ there has been some concern of postoperative cognitive disturbances,^15–18^ which have the potential to impact everyday functioning and quality of life. Studies suggest a central role of the thalamus in cognition,^19,20^ with the Vim in particular implicated in speech and language abilities.^21–23^ Based on these findings, thalamotomy might be expected to negatively impact speech and language abilities. However, findings on the effects of thalamotomy across cognitive domains have been mixed. For example, worsening cognition has been reported across domains of processing speed, executive function, memory and verbal fluency at the group^16–18,24^ and individual level.^25^ However, there are also reports of stable or even improved postoperative cognitive performance in these same domains.^26,27^ One difficulty in interpreting these findings is that most studies have small sample sizes and, consequently, low statistical power to detect significant cognitive changes.^28^

With the increasing popularity of non-invasive thalamotomy procedures for tremor, particularly MRgFUS, there is a need to document the scope of cognitive decline following thalamotomy. The aim of the present meta-analysis was to provide a cross-study summary of the impact of Vim thalamotomy for tremor across multiple cognitive domains. We examined a wide range of cognitive domains given that previous studies have found mixed findings across domains. In secondary analyses, we examined whether MRgFUS is associated with a more favourable postoperative cognitive profile given its more precise targeting.^12^

## Materials and methods

The present study was conducted in accordance with the Preferred Reporting Items for Systematic Reviews and Meta-Analyses (PRISMA) guidelines^29^ and submitted to PROSPERO (CRD42021241872) on 9 March 2021, with registration confirmation on 9 April 2021. We searched MEDLINE, Embase Classic, Embase and EBM Reviews for relevant studies on 12 February 2021 and again on 9 May 2022. Search strategies were developed in collaboration with a health sciences librarian (see Supplemental Material for details of the search strategy). The search was restricted to English, limited to human studies and had no restriction on publication date. In addition, we manually searched the reference lists of reviewed papers for relevant papers not identified in the literature search. Covidence software^30^ was used to review papers, identify duplicates and track screening decisions. Two raters independently screened titles and abstracts. Full texts were retrieved for relevant papers and inclusion criteria were independently assessed by two reviewers. Inconsistencies were resolved by consensus.

Inclusion criteria for the individual studies were as follows: (i) original peer-reviewed research; (ii) unilateral Vim thalamotomy; (iii) at least one validated cognitive test at baseline and follow-up; (iv) follow-up testing occurring at least one month after treatment; and (v) randomized controlled trials, open-label trials or case series with ≥ 3 participants. For studies reporting on overlapping groups of participants, the study that included a greater number of participants was included. Exclusion criteria were as follows: (i) case reports, review articles, editorials, letters and conference abstracts; (ii) targets other than the Vim or combined targets; (iii) studies that did not assess cognition; and (iv) thalamic lesions that were secondary to stroke or tumour.

Two authors independently extracted the following information from eligible studies: (i) study characteristics (authors, year of publication, study centre); (ii) patient characteristics (e.g. age, sex, diagnosis, age at tremor onset); (iii) sample size; (iv) surgical technique; (v) side of lesion; (vi) months between treatment and follow-up; (vii) number of patients with baseline cognitive impairment; and (viii) means and standard deviations (SD) of pre- and postoperative test scores. When other units were reported (e.g. median and interquartile range), the mean and SD were estimated.^31^

Neuropsychological tests were categorized into seven cognitive domains based on widely used definitions,^32^ including global cognition, verbal memory, non-verbal memory, executive function, phonemic fluency, semantic fluency and visuospatial processing. For verbal and non-verbal memory domains, we examined immediate and delayed recall separately. Each test was assigned to only one cognitive domain under the supervision of a clinical neuropsychologist (J.S.R.). When studies included more than one test per domain, the most representative or consistently reported test across studies was selected for the meta-analysis (See Supplementary Table 1). A minimum of three studies were required to perform a meta-analysis of any given cognitive domain in order to reliably estimate effects.

### Statistical analyses

In primary analyses, we examined changes in cognition across all thalamotomy techniques. In secondary analyses, we restricted the analyses to studies using MRgFUS given its more precise lesioning. There were insufficient data (i.e. < 3 studies per cognitive domain) to perform separate analyses for radiofrequency ablation and Gamma Knife radiosurgery. All statistical analyses were performed using the metafor package^33^ in R (version 4.0.2). We calculated standardized mean differences (SMDs) as Hedges’ g and 95% confidence intervals (CIs) of the change between pre- and postoperative cognitive scores. A negative SMD indicated postoperative decline, whereas a positive SMD reflected postoperative improvement. Pooling of SMDs across studies was performed using a random-effects model and the DerSimonian and Laird method.^34^ Heterogeneity across studies was assessed using Cochrane’s Q test (statistical significance set at *P* < 0.10) and I^2^ statistics.^35^ With respect to I^2^, values of 25%, 50% and 75% were deemed small, moderate and large heterogeneity, respectively.^36^

### Risk of bias and quality assessment

Risk of bias was assessed using the National Institute of Health (NIH) quality assessment tool for before–after (pre–post) studies with no control group.^37,38^ The tool includes 12 questions that assess the internal validity of a study. Questions ask about eligibility/selection criteria, the representativeness of study participants to the clinical population of interest, sample size, blinding of examiners and the proportion of participants lost to follow-up. Questions are assigned one of the following response options: ‘yes’, ‘no’, ‘cannot determine’, ‘not applicable’ or ‘not reported’. Studies are assigned an overall rating of ‘good', ‘fair' or ‘poor'. Two reviewers performed the assessments independently, and any disagreements were discussed and resolved. Given the low number of studies included in the present meta-analysis, publication bias was not assessed.^39^

The GRADEpro Guideline Development Tool was used to assess the quality of evidence for each cognitive domain.^40^ The Grading of Recommendations Assessment, Development and Evaluation (GRADE) criteria considers study design, risk of bias, inconsistency, indirectness, imprecision and publication bias. Based on these variables, the software generates a GRADE rating of high, moderate, low, or very low to reflect the certainty of the reported effect.

### Data availability

All data are available in the Supplementary material.

## Results

**Figure 1:**
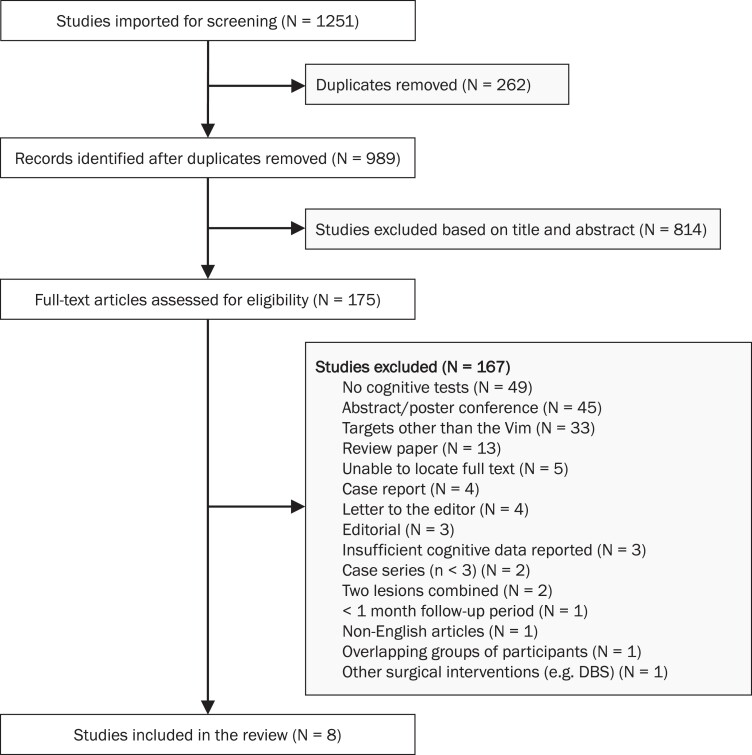
Flow diagram showing the search and selection procedure for the meta-analysis.

The results of the search and article-selection process are summarized in Fig. 1. Of the 1251 records reviewed, eight studies met inclusion criteria. The most common reason studies were excluded was because cognition was not assessed. The characteristics of the eight included studies are shown in Table 1. Across studies, there were 198 participants (mean age = 66.10, SD: 4.68); 94 participants with essential tremor, 99 participants with Parkinson’s disease and 5 participants with multiple sclerosis. Most of the participants were male. A total of 72 participants were treated with MRgFUS,^18,25,27,41^ 76 with radiofrequency^16,17,26^ and 50 with Gamma Knife radiosurgery.^24^ Of the total sample, 147 underwent left-sided thalamotomy and 51 underwent right-sided thalamotomy. Across studies, follow-up testing occurred between 1–12 months after thalamotomy. Of the 198 participants, only 193 underwent neuropsychological testing and were included in the meta-analysis.

**Table 1:** Study characteristics

Study	Surgical technique	Clinical diagnosis	Left Vim, *n* (%)	Right Vim, *n* (%)	Mean age (years)	Male *n* (%)	Mean tremor duration (years)	Baseline cognitive impairment, *n* (%)	Baseline cognitive exclusion criteria	Follow-up period (months)
Fukuda *et al.*^26^	RF	PD = 13	6 (46.2%)	7 (53.8%)	63.2	5 (38.5%)	NR	NR	NR	1
Gasca-Salas *et al.*^27^	MRgFUS	ET = 23	22 (95.7%)	1 (4.3%)	64.1	17 (73.9%)	16.6	10 (43.5%)	NR	12
Jung *et al.*^25^	MRgFUS	ET = 20	20 (100%)	0 (0%)	64.1	17 (85%)	21.2	9 (45.0%)	NR	> 6
Martínez-Fernández *et al.*^41^	MRgFUS	ET = 9	8 (88.9%)	1 (11.1%)	71.0	5 (56%)	31.0	NR	NR	NR
Nijhawan *et al.*^16^	RF	PD = 31	22 (71.0%)	9 (29.0%)	62.8	25 (80.7%)	6.6	NR	NR	5.6
Schuurman *et al.*^17^	RF	ET = 6 PD = 21 MS = 5 Total = 32	11 (34.4%)	21 (65.6%)	63	17 (53.1%)	12.9	NR	MMSE < 24	6
Sperling *et al.*^18^	MRgFUS	PD = 20	20 (100%)	0 (0%)	NR	NR	NR	NR	MoCA ≤ 21	3
Witjas *et al.*^24^	GKRS	ET = 36 PD = 14 Total = 50^a^	38 (76%)	12 (24%)	74.5	32 (64%)	22.4	NR	NR	12

^a^ only 45 of the 50 participants completed neuropsychological testing. ET = essential tremor, GKRS = Gamma Knife radiosurgery, MMSE = Mini-Mental State Examination, MoCA = Montreal Cognitive Assessment, MRgFUS = magnetic resonance-guided focused ultrasound, MS = multiple sclerosis, NR = not reported, PD = Parkinson’s disease, RF = radiofrequency, Vim = ventral intermediate nucleus of the thalamus.

**Figure 2:**
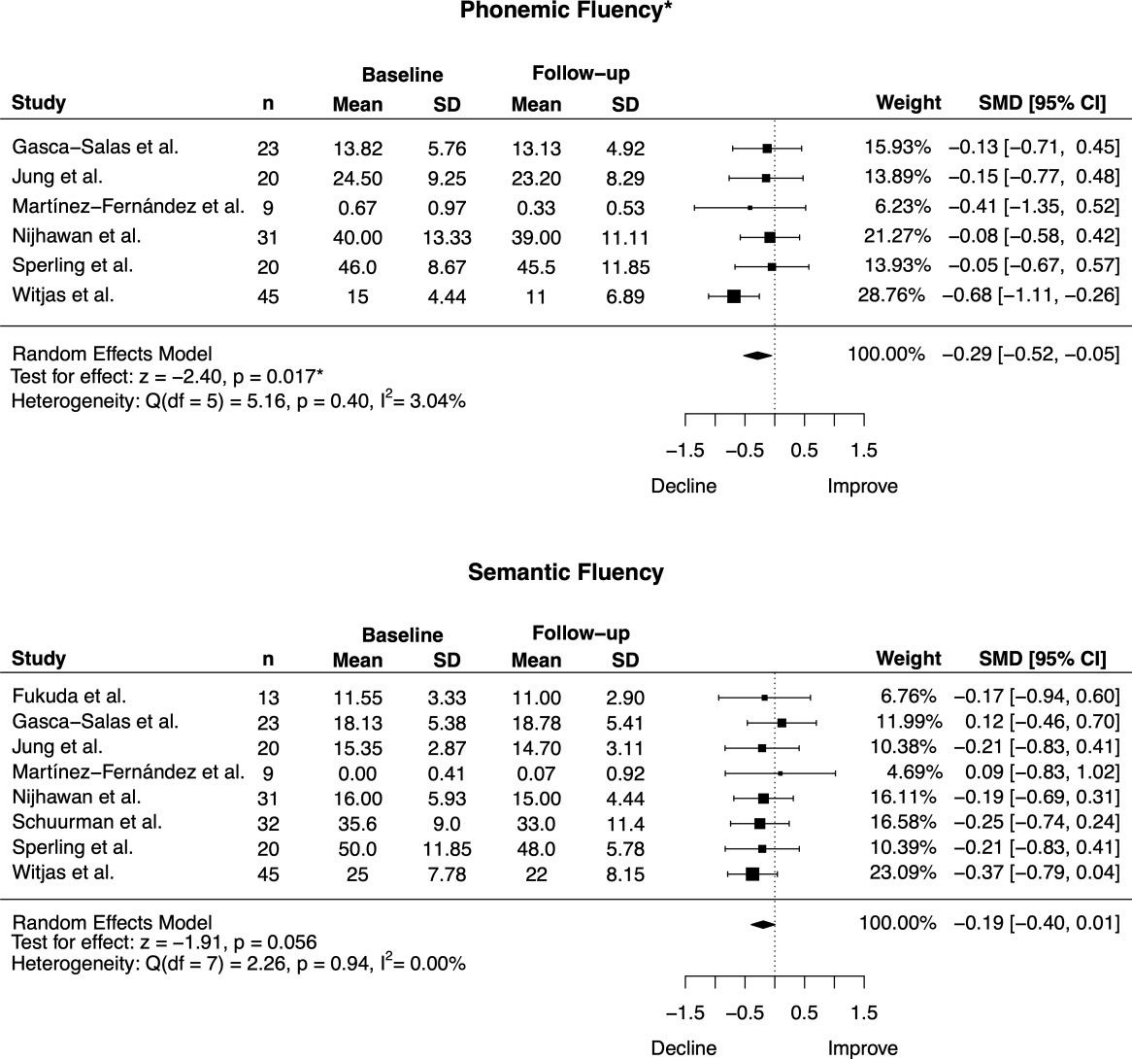
**Forest plots depicting the effect of thalamotomy on phonemic and semantic fluency across all surgical techniques.** There was a significant postoperative decline for phonemic fluency and a trend toward a significant decline for semantic fluency. *n* = sample size, SD = standard deviation, SMD = standardized mean difference, CI = confidence interval. *P < 0.05.

Across all surgical techniques, there was a small postoperative decline in phonemic fluency [SMD = −0.29, 95% CI = (−0.52, −0.05), *P* = 0.017; heterogeneity: Q = 5.16, *P* = 0.40, I^2^ = 3.04%] and a trend in the same direction for semantic fluency [SMD = −0.19, 95% CI = (−0.40, 0.01), *P* = 0.056; heterogeneity: Q = 2.26, *P* = 0.94, I^2^ = 0.00%; Fig. 2 and Table 2). No significant postoperative changes were observed in the remaining cognitive domains, including global cognition, verbal and non-verbal memory, executive function and visuospatial processing (SMDs ranged from −0.10 to 0.17, *P* values > 0.14; Table 2 and Supplementary Fig. 1). For all cognitive domains, statistical heterogeneity was extremely small and non-significant (*P* > 0.40; I^2^ ranged from 0% to 3.04%; Table 2).

**Table 2:** Effect of thalamotomy on cognition across all surgical techniques

Domain	Studies included	*n*	SMD	95% CI	*P*-value	Heterogeneity
Global cognition	Fukuda *et al.*^26^; Jung *et al.*^25^; Sperling *et al.*^18^; Witjas *et al.*^5^	98	−0.10	−0.38, 0.18	0.48	Q = 0.74, *P* = 0.86; I^2^ = 0.00%
Executive function	Gasca-Salas *et al.*^27^; Jung *et al.*^25^; Martínez-Fernández *et al.*^41^; Nijhawan *et al.*^16^; Schuurman *et al.*^17^; Sperling *et al.*^18^	129	−0.09	−0.33, 0.15	0.48	Q = 4.38, *P* = 0.48; I^2^ = 0.00%
Phonemic fluency	Gasca-Salas *et al.*^27^; Jung *et al.*^25^; Martínez-Fernández *et al.*^41^; Nijhawan *et al.*^16^; Sperling *et al.*^18^; Witjas *et al.*^5^	148	−0.29	−0.52, −0.05	0.017*	Q = 5.16, *P* = 0.40; I^2^ = 3.04%
Semantic fluency	Fukuda *et al.*^26^; Gasca-Salas *et al.*^27^; Jung *et al.*^25^; Martínez-Fernández *et al.*^41^; Nijhawan *et al.*^16^; Schuurman *et al.*^17^; Sperling *et al.*^18^; Witjas *et al.*^5^	193	−0.19	−0.40, −0.01	0.056	Q = 2.26, *P* = 0.94; I^2^ = 0.00%
Verbal memory: immediate recall	Fukuda *et al.*^26^; Gasca-Salas *et al.*^27^; Jung *et al.*^25^; Martínez-Fernández *et al.*^41^; Nijhawan *et al.*^16^; Schuurman *et al.*^17^; Sperling *et al.*, 2018	146	0.17	−0.06, 0.40	0.14	Q = 0.78, *P* = 0.99; I^2^ = 0.00%
Verbal memory: delayed recall	Gasca-Salas *et al.*^27^; Jung *et al.*^25^; Martínez-Fernández *et al.*^41^; Nijhawan *et al.*^16^; Schuurman *et al.*^17^; Sperling *et al.*^18^	133	0.09	−0.15, 0.33	0.47	Q = 4.21, *P* = 0.52; I^2^ = 0.00%
Non-verbal memory: immediate recall	Jung *et al.*^25^; Nijhawan *et al.*^16^; Schuurman *et al.*^41^; Sperling *et al.*^18^	98	0.12	−0.15, 0.40	0.40	Q = 1.52, *P* = 0.68; I^2^ = 0.00%
Non-verbal memory: delayed recall	Jung *et al.*^25^; Schuurman *et al.*^41^; Sperling *et al.*^18^	72	0.15	−0.18, 0.47	0.38	Q = 0.67, *P* = 0.71; I^2^ = 0.00%
Visuospatial processing	Fukuda *et al.*^26^; Gasca-Salas *et al.*^27^; Jung *et al.*^25^; Schuurman *et al.*^41^	88	0.00	−0.29, 0.30	0.99	Q = 0.83, *P* = 0.84; I^2^ = 0.00%

* *P* < 0.05. *n* = sample size, SMD = standardized mean difference, CI = confidence interval.

In secondary analyses, we examined whether MRgFUS is associated with a more favourable postoperative cognitive profile compared with the other thalamotomy techniques (i.e. radiofrequency and Gamma Knife radiosurgery) given its more precise lesioning. Four of the included studies used MRgFUS and at least three of these studies examined executive function, phonemic fluency, semantic fluency, verbal memory and visuospatial processing. In this analysis, there were no significant postoperative changes in any of the cognitive domains examined (SMDs ranged from −0.14 to 0.15, *P* > 0.37; Table 3 and Supplementary Fig. 2). Most notably, unlike the primary analysis, we did not observe a significant decline in phonemic fluency [SMD = −0.14, 95% CI: (−0.47, 0.18), *P* = 0.39] or semantic fluency [SMD = −0.07, 95% CI: (−0.39, 0.26), *P* = 0.69].

**Table 3:** Effect of magnetic resonance-guided focused ultrasound thalamotomy on cognition by domain

Domain	Studies included	*n*	SMD	95% CI	*P*-value	Heterogeneity
Executive function	Gasca-Salas *et al.*^27^; Jung *et al.*^25^; Martínez-Fernández *et al.*^41^; Sperling *et al.*^18^	72	0.08	−0.25, 0.41	0.64	Q = 1.55, *P* = 0.67; I^2^ = 0.00%
Phonemic fluency	Gasca-Salas *et al.*^27^; Jung *et al.*^25^; Martínez-Fernández *et al.*^41^; Sperling *et al.*^18^	72	−0.14	−0.47, 0.18	0.39	Q = 0.42, *P* = 0.94; I^2^ = 0.00%
Semantic fluency	Gasca-Salas *et al.*^27^; Jung *et al.*^25^; Martínez-Fernández *et al.*^41^; Sperling *et al.*^18^	72	−0.07	−0.39, 0.26	0.69	Q = 0.93, *P* = 0.82; I^2^ = 0.00%
Verbal memory: immediate recall	Gasca-Salas *et al.*^27^; Jung *et al.*^25^; Martínez-Fernández *et al.*^41^; Sperling *et al.*^18^	72	0.15	−0.18, 0.48	0.37	Q = 0.69, *P* = 0.88; I^2^ = 0.00%
Verbal memory: delayed recall	Gasca-Salas *et al.*^27^; Jung *et al.*^25^; Martínez-Fernández *et al.*^41^; Sperling *et al.*^18^	72	0.15	−0.23, 0.54	0.43	Q = 3.94, *P* = 0.27; I^2^ = 23.92%
Visuospatial processing	Gasca-Salas *et al.*^27^; Jung *et al.*^25^; Martínez-Fernández *et al.*^41^	52	0.05	−0.34, 0.43	0.81	Q = 1.56, *P* = 0.46; I^2^ = 0.00%

*n* = sample size, SMD = standardized mean difference, CI = confidence interval.

Due to the limited number of studies included in the present study (*n* = 8), along with their study characteristics, we had insufficient data to perform additional subgroup analyses, such as those comparing left versus right-sided thalamotomy or by clinical diagnosis.^42^

Risk of bias was assessed using the NIH tool for pre–post studies. According to this tool, five studies were deemed to be ‘good’ in quality and three studies were deemed to be ‘fair’. No studies were deemed to be ‘poor’ in quality. The main reason for assigning a ‘fair’ rating was because of the small sample size and studies having relatively high dropout rates after baseline neuropsychological testing (Supplementary Table 2). As for the quality of the evidence, according to GRADEpro guidelines, the certainty of the effect for all cognitive domains was low (Supplementary Table 3). Certainty was downgraded due to the lack of a control group and imprecision of included studies (i.e. small sample size).

## Discussion

Thalamotomy is an effective treatment for tremor, however patients often have concerns about cognitive deterioration. The goal of the present study was to quantitatively summarize the impact of unilateral Vim thalamotomy for tremor across multiple cognitive domains in a sample of patients with essential tremor, Parkinson’s disease or multiple sclerosis. We found a small significant postoperative decline in phonemic fluency and a trend in the same direction for semantic fluency. No significant postoperative changes were observed in other domains, including global cognition, verbal and non-verbal memory, executive function and visuospatial processing. When we restricted the analyses to studies using MRgFUS, there was no evidence of postoperative decline in any cognitive domain. Taken together, the present study suggests that unilateral Vim thalamotomy, and particularly MRgFUS, is relatively safe from a cognitive standpoint.

The main finding of the study was that there were no substantial cognitive changes following unilateral Vim thalamotomy in analyses that collapsed across all thalamotomy techniques. The only cognitive domain to show a significant postoperative decline was verbal fluency. This decline was small in magnitude and therefore may have minimal impact on daily functioning and quality of life.^43^ However, mild declines in verbal fluency might be consequential to individuals with pre-existing cognitive deficits. Given that individuals with essential tremor, Parkinson’s disease and multiple sclerosis often present with cognitive difficulties,^44,45^ the clinical relevance of this decline is an important area for future research. Future thalamotomy studies examining postoperative cognition should include measures of functional capacity and quality of life to determine whether changes in cognition are relevant to patients’ daily lives.

A related issue to consider is whether baseline cognitive impairment increases the risk of postoperative cognitive decline. The current thalamotomy literature addressing this question is limited and has produced mixed findings.^25,27^ Most of the studies included in our meta-analysis did not report cognitive diagnoses at baseline. This is a critical question that should be addressed in future research.

Our finding of a small selective decline in verbal fluency is consistent with several Vim deep brain stimulation studies, which also report postoperative decline in the same domain.^43,46,47^ The mechanism by which Vim thalamotomy selectively impacts verbal fluency is not clear. Data from functional neuroimaging, neurophysiological and focal lesion studies suggest a role for the dominant (typically left) thalamus, and specifically the Vim, in language abilities.^22,23,48^ The thalamus has also been implicated in speech motor control,^49^ and therefore, the decline in verbal fluency might reflect slowed speech rather than changes in language per se. To tease these possibilities apart, future thalamotomy studies should include speech or articulatory tasks in addition to verbal fluency measures.

An important finding was that when we restricted the analyses to thalamotomy studies using MRgFUS, no postoperative cognitive changes were observed across any domain, including phonemic and semantic fluency. Preserved cognition following MRgFUS might be due to the generation of smaller, more precise lesions, which is made possible with real-time monitoring of the lesion and thermographic feedback.^13,50^ It could be argued that we lacked the power to detect cognitive changes because only four studies were included in this analysis. However, the effect sizes (which are independent of sample size) for phonemic and semantic fluency in the MRgFUS analysis were considerably smaller than those in the primary analysis (phonemic fluency: −0.14 versus −0.29; semantic fluency: −0.07 versus—0.19). There were insufficient data to perform separate analyses for radiofrequency ablation and Gamma Knife radiosurgery. However, it is worth noting that the study using Gamma Knife radiosurgery showed the largest magnitude of postoperative decline for phonemic and semantic fluency (SMD = −0.68 and SMD = −0.37, respectively).^24^

Previous work demonstrates that lesion location, volume and extent are important factors associated with the rate of adverse effects.^50,51^ The posterior portion of the Vim has been identified as the area of optimal tremor response and lesions extending beyond this area have been associated with side effects, including speech disturbances.^50,51^ Lesioning procedures require a balance between lesion size and risk of adverse effects since larger lesions are more likely to achieve maximum tremor benefit but have a greater likelihood of side effects.^50^ Gamma Knife thalamotomy might result in larger than expected lesions given the potentially progressive nature of radiation-induced tissue injury.^52^ It would be beneficial for future work to perform postoperative MRI lesion analyses to help determine the optimal lesion location and volume that maximize long-term therapeutic outcomes, while minimizing cognitive disturbances.

There are several limitations to this study. The main limitation is that only eight studies were included in the primary meta-analysis and only four studies were included in the MRgFUS analysis. In addition, not all studies examined all cognitive domains of interest. While analyses for phonemic and semantic fluency had larger sample sizes, other cognitive domains, such as visuospatial processing, had smaller sample sizes. As such, these findings should be interpreted with some caution. Second, our meta-analysis compared pre- and postoperative test performance, since most studies lacked a control group. As a result, practice effects may have inflated postoperative test scores,^53^ potentially underestimating the extent of verbal fluency decline and/or masking decline in other cognitive domains. Third, our meta-analysis included several patient groups (Parkinson’s disease, essential tremor and multiple sclerosis) and we did not have sufficient data to perform subgroup analyses by clinical diagnosis. Measures of statistical heterogeneity were exceptionally low, suggesting consistent effects of thalamotomy on cognition across diagnostic groups. Finally, as with all meta-analyses, the quality is limited by the number and the level of the included studies. In terms of risk of bias, five of the eight studies were deemed to be of ‘good’ quality and three studies were deemed to be of ‘fair’ quality. According to GRADEpro guidelines, the certainty of the effect for all cognitive domains was low.

In summary, our meta-analysis provides the first cross-study evidence that unilateral Vim thalamotomy for tremor is relatively safe from a cognitive standpoint, however, there may be a small postoperative decline in verbal fluency. The cognitive safety profile of MRgFUS may be superior to other thalamotomy techniques, although this needs to be confirmed in future work. There is a need for more well-powered studies investigating the cognitive effects of unilateral and bilateral MRgFUS Vim thalamotomy for tremor given its growing popularity. Future studies should determine whether the side of thalamotomy (left versus right thalamotomy) or specific baseline factors (e.g. pre-existing cognitive impairment or clinical diagnosis) influence cognitive outcomes.

## Supplementary Material

fcac287_Supplementary_Data

## Abbreviations

CI =: confidence interval
GRADE =: Grading of Recommendations Assessment, Development and Evaluation
MRgFUS =: magnetic resonance-guided focused ultrasound
NIH =: National Institute of Health
PRISMA =: Preferred Reporting Items for Systematic Reviews and Meta-Analyses
SD =: standard deviation
SMD =: standardized mean difference
Vim =: ventral intermediate nucleus of the thalamus
