# Supplementary material for: Cognitive effects of unilateral thalamotomy for tremor: a meta-analysis
Source: Brain Commun. 2022 Nov 4;4(6):fcac287. doi: 10.1093/braincomms/fcac287 (PMC9683603; doi:10.1093/braincomms/fcac287)
Supplement: fcac287_Supplementary_Data [file fcac287_supplementary_data.pdf]

Supplementary Material

Search strategy 2
Supplementary Table 1: Pre- and post-thalamotomy outcomes by cognitive domain 5
Supplementary Fig. 1: Forest plots depicting the effect of thalamotomy on cognition across all surgical techniques . 7
Supplementary Fig. 2: Forest plots depicting the effect of magnetic resonance-guided focused ultrasound thalamotomy on cognition 10
Supplementary Table 2: Risk of bias 12
Supplementary Table 3: GRADEpro quality of evidence assessment 14

Search Strategy

Database: Ovid MEDLINE(R) and Epub Ahead of Print, In-Process & Other Non-Indexed Citations, Daily and Versions(R) <1946 to May 06, 2022> Search Strategy:

----

1 exp Thalamus/su [Surgery] (2107)
2 thalamotom*.mp. (1284)
3 ((thalamus or thalamic or ventral thalamic nucle*) adj3 (surg* or stereotactic or neurosurg* or operat* or procedure* or technique* or shunt*)).mp. (445)
4 or/1-3 (2900)
5 exp Cognition/ (186022)
6 exp Cognition Disorders/ (109363)
7 (cognit* or neurocognit*).mp. (527026)
8 exp Memory/ (149398)
9 exp Memory Disorders/ (32022)
10 memory.mp. (318432)
11 exp Language/ (230671)
12 exp Speech/ (36756)
13 exp Speech Disorders/ (32152)
14 (language* or speech or verbal or linguistic*).mp. (391452)
15 exp Executive Function/ (18313)
16 executive function*.mp. (39335)
17 exp Attention/ (84596)
18 attention.mp. (540526)
19 exp Space Perception/ (63166)
20 visuospatial.mp. (11318)
21 exp Neuropsychology/ (2546)
22 neuropsycholog*.mp. (131937)
23 or/5-22 (173851)
24 4 and 23 (370)
25 limit 24 to english language (322)
26 from 25 keep 1-306 (322)

****

Database: Embase Classic+Embase <1947 to 2022 Week 18> Search Strategy:

----

1 exp thalamotomy/ (2182)
2 exp thalamus/su [Surgery] (748)
3 thalamotom*.mp. (2705)
4 ((thalamus or thalamic or ventral thalamic nucle*) adj3 (surg* or stereotactic or neurosurg* or operat* or procedure* or technique* or shunt*)).mp. (624)
5 or/1-4 (3795)
6 exp cognition/ (2793163)
7 exp cognitive defect/ (563699)
8 exp cognition assessment/ (29126)

9 (cognit* or neurocognit*).mp. (810212)
10 exp memory/ (340858)
11 exp memory disorder/ (88002)
12 memory.mp. (455632)
13 exp language/ (145682)
14 exp language disability/ (75521)
15 exp speech/ (118395)
16 exp speech disorder/ (110782)
17 (language* or speech or verbal or linguistic*).mp. (548109)
18 executive function/ (48834)
19 executive function*.mp. (67588)
20 exp attention/ (304720)
21 attention.mp. (710432)
22 exp depth perception/ (27361)
23 visuospatial.mp. (15876)
24 exp neuropsychology/ (20070)
25 neuropsycholog*.mp. (129572)
26 or/6-25 (4192116)
27 5 and 26 (982)
28 limit 27 to english language (893)

****

Database: EBM Reviews - Cochrane Central Register of Controlled Trials <April 2022 > Search Strategy:

----

1 exp Thalamus/su [Surgery] (7)
2 thalamotom*.mp. (67)
3 ((thalamus or thalamic or ventral thalamic nucle*) adj3 (surg* or stereotactic or neurosurg* or operat* or procedure* or technique* or shunt*)).mp. (88)
4 or/1-3 (149)
5 exp Cognition/ (11768)
6 exp Cognition Disorders/ (5975)
7 (cognit* or neurocognit*).mp. (92785)
8 exp Memory/ (8274)
9 exp Memory Disorders/ (1259)
10 memory.mp. (27256)
11 exp Language/ (5682)
12 exp Speech/ (921)
13 exp Speech Disorders/ (942)
14 (language* or speech or verbal or linguistic*).mp. (36859)
15 exp Executive Function/ (1267)
16 executive function*.mp. (7371)
17 exp Attention/ (5825)
18 attention.mp. (36026)
19 exp Space Perception/ (2921)

20 visuospatial.mp. (1253)
21 exp Neuropsychology/ (22)
22 neuropsycholog*.mp. (13570)
23 or/5-22 (160406)
24 4 and 23 (34)
25 limit 24 to english language (32)

****

Supplementary Table 1: Pre- and post-thalamotomy outcomes by cognitive domain

Cognitive Domain	Test	Study	n	Baseline Mean (SD)	Follow-Up Mean (SD)	P-Value	Trend
Global cognition	HDS-R	Fukuda et al., 2000	13	23.36 (4.11)	24.00 (4.29)	0.24	Improve
	K-MMSE	Jung et al., 2018	20	28.70 (1.45)	28.50 (1.28)	0.36	Decline
	MDRS	Witjas et al., 2015	45	135 (3.70)	134 (5.93)	>0.05	Decline
	MoCA	Sperling et al., 2018	20	25.5 (3.33)	25.5 (2.59)	N/A	Stable
Executive function (inhibition)	Stroop color-word (inhibition condition)	Gasca-Salas et al., 2019	23	67.08 (15.71) [I]	67.15 (17.23) [I]	0.98	Decline
		Jung et al., 2018	20	83.80 (21.69)	85.00 (24.48)	0.63	Improve
		Martínez-Fernández et al., 2021	9	-0.07 (0.76)	-0.33 (0.91)	N/A	Decline
		Nijhawan et al., 2009	25	-0.76 (1.93)	-0.98 (1.07)	>0.05	Decline
		Schuurman et al., 2002	32	131.1 (41.0) [I]	153.3 (59.7) [I]	<0.05	Decline*
		Sperling et al., 2018	20	46 (8)	48.5 (4.81)	N/A	Improve
Phonemic fluency	FAS	Nijhawan et al., 2009	31	40 (13.33)	39 (11.11)	0.035	Decline*
		Sperling et al., 2018	20	46 (8.67)	45.5 (11.85)	N/A	Decline
	Unspecified	Gasca-Salas et al., 2019	23	13.82 (5.76)	13.13 (4.92)	0.45	Decline
		Jung et al., 2018	20	24.50 (9.25)	23.20 (8.29)	0.84	Decline
		Martínez-Fernández et al., 2021	9	0.67 (0.97)	0.33 (0.53)	N/A	Decline
		Witjas et al., 2015	45	15 (4.44)	11 (6.89)	>0.05	Decline
Semantic fluency	Animals	Fukuda et al., 2000	13	11.55 (3.33)	11 (2.90)	0.49	Decline
		Jung et al., 2018	20	15.35 (2.87)	14.70 (3.11)	0.33	Decline
		Nijhawan et al., 2009	31	16 (5.93)	15 (4.44)	>0.05	Decline
		Sperling et al., 2018	20	50 (11.85)	48 (5.78)	N/A	Decline
	Animals, occupations	Schuurman et al., 2002	32	35.6 (9.0)	33.0 (11.4)	>0.05	Decline
	Unspecified	Gasca-Salas et al., 2019	23	18.13 (5.38)	18.78 (5.41)	0.33	Improve
		Martínez-Fernández et al., 2021	9	0.00 (0.41)	0.07 (0.92)	N/A	Improve
		Witjas et al., 2015	45	25 (7.78)	22 (8.15)	<0.025	Decline*
Verbal memory: immediate recall	AMIPB	Nijhawan et al., 2009	29	-0.80 (1.07)	-0.52 (1.24)	>0.05	Improve
	AVLT	Fukuda et al., 2000	13	29.18 (14.00)	31.27 (12.98)	0.22	Improve
		Schuurman et al., 2002	32	31.1 (10.1)	32.8 (10.3)	>0.05	Improve

	CERAD	Gasca-Salas et al., 2019	23	7.87 (1.46)	7.87 (1.22)	0.61	Stable
		Martínez-Fernández et al., 2021	9	7.40 (2.30)	8.20 (0.84)	N/A	Improve
	HVLT-R	Sperling et al., 2018	20	44.5 (10.96)	47 (10.89)	N/A	Improve
	SVLT	Jung et al., 2018	20	21.35 (4.97)	22.00 (5.67)	0.45	Improve
Verbal memory: delayed recall	AMIPB	Nijhawan et al., 2009	29	-0.68 (0.85)	-0.69 (0.73)	>0.05	Decline
	AVLT	Schuurman et al., 2002	32	6.1 (2.5)	6.3 (3.5)	>0.05	Improve
	CERAD	Gasca-Salas et al., 2019	23	6.30 (2.55)	5.91 (2.23)	0.59	Decline
		Martínez-Fernández et al., 2021	9	5.20 (3.03)	6.20 (1.92)	N/A	Improve
	HVLT-R	Sperling et al., 2018	20	43 (7.78)	48.5 (9.41)	N/A	Improve
	SVLT	Jung et al., 2018	20	6.35 (2.76)	6.15 (3.05)	0.75	Decline
Non-verbal memory: immediate recall	BVMT-R	Sperling et al., 2018	20	42.5 (14.07)	41.5 (12.37)	N/A	Decline
	RCFT	Jung et al., 2018	20	19.30 (6.18)	22.05 (7.78)	0.049	Improve*
	RMT faces	Nijhawan et al., 2009	26	8.00 (4.63)	9.00 (3.89)	>0.05	Improve
	WMS-R (visual reproduction)	Schuurman et al., 2002	32	52.1 (40.3)	51.5 (36.7)	>0.05	Decline
Non-verbal memory: delayed recall	BVMT-R	Sperling et al., 2018	20	44.5 (12.96)	46.5 (13.48)	N/A	Improve
	RCFT	Jung et al., 2018	20	18.85 (5.31)	21.13 (7.25)	0.038	Improve*
	WMS-R (visual reproduction)	Schuurman et al., 2002	32	51.5 (37.6)	52.2 (34.4)	>0.05	Improve
Visuospatial processing	JLO	Gasca-Salas et al., 2019	23	25.46 (3.93)	24.69 (3.43)	0.4	Decline
		Martínez-Fernández et al., 2021	9	0.07 (0.64)	0.53 (1.22)	N/A	Improve
	RCFT	Jung et al., 2018	20	34.05 (1.54)	34.30 (1.58)	0.62	Improve
	WAIS-R (PIQ)	Fukuda et al., 2000	13	84.77 (9.01)	86.08 (10.81)	0.41	Improve
	WAIS-R (object assembly)	Schuurman et al., 2002	32	8.6 (2.7)	8.6 (2.7)	>0.05	Stable

Note. * $p < 0.05$; [I] = higher scores represent worse performance (these scores were inverted for the meta-analysis). Abbreviations: AMIPB = Adult Memory and Information Processing Battery, AVLT = Rey Auditory Verbal Learning Test, BVMT-R = Brief Visuospatial Memory Test-Revised, CERAD = Consortium to Establish a Registry for Alzheimer's Disease, HDS-R = Revised Hasegawa's Dementia Scale, HVLT-R = Hopkins Verbal Learning Test-Revised, JLO = Judgment of Line Orientation, K-MMSE = Korean Mini-Mental State Examination, MDRS = Mattis Dementia Rating Scale, MoCA = Montreal Cognitive Assessment, RCFT = Rey Complex Figure Test and Recognition Trial, RMT = Recognition Memory Test, SVLT = Shiraz Verbal Learning Test, WAIS-R = Wechsler Adult Intelligence Scale, WMS-R = Wechsler Memory Scale Revised.

Supplementary Fig. 1: Forest plots depicting the effect of thalamotomy on cognition across all surgical techniques

Global Cognition

Study	n	Baseline Mean	Baseline SD	Follow-up Mean	Follow-up SD	Weight	SMD [95% CI]
Fukuda et al., 2000	13	23.36	4.11	24.00	4.29	13.27%	0.15 [−0.62, 0.92]
Jung et al., 2018	20	28.70	1.45	28.50	1.28	20.42%	−0.14 [−0.76, 0.48]
Sperling et al., 2018	20	25.5	3.33	25.5	2.59	20.47%	0.00 [−0.62, 0.62]
Witjas et al., 2015	45	135	3.70	134	5.93	45.83%	−0.20 [−0.61, 0.21]
Random Effects Model						100.00%	−0.10 [−0.38, 0.18]

Test for effect: z = −0.71, p = 0.48

Heterogeneity: Q(df = 3) = 0.74, p = 0.86, I^2= 0.00%

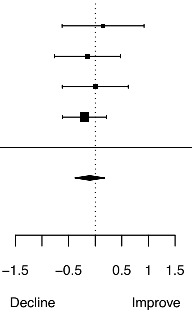

Executive Functioning (Inhibition)

Study	n	Baseline Mean	Baseline SD	Follow-up Mean	Follow-up SD	Weight	SMD [95% CI]
Gasca–Salas et al., 2019	23	−67.08	15.71	−67.15	17.23	17.63%	−0.00 [−0.58, 0.57]
Jung et al., 2018	20	83.80	21.69	85.00	24.48	15.33%	0.05 [−0.57, 0.67]
Martínez–Fernández et al., 2021	9	−0.07	0.76	−0.33	0.91	6.82%	−0.30 [−1.22, 0.63]
Nijhawan et al., 2009	25	−0.76	1.93	−0.98	1.07	21.17%	−0.14 [−0.66, 0.39]
Schuurman et al., 2002	32	−131.1	41.0	−153.3	59.7	23.98%	−0.43 [−0.92, 0.07]
Sperling et al., 2018	20	46.0	8.00	48.5	4.81	15.07%	0.37 [−0.25, 1.00]
Random Effects Model						100.00%	−0.09 [−0.33, 0.15]

Test for effect: z = −0.71, p = 0.48

Heterogeneity: Q(df = 5) = 4.38, p = 0.50, I^2= 0.00%

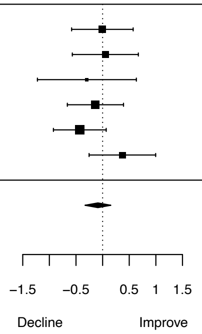

Phonemic Fluency*

Study	n	Baseline Mean	Baseline SD	Follow-up Mean	Follow-up SD	Weight	SMD [95% CI]
Gasca–Salas et al., 2019	23	13.82	5.76	13.13	4.92	15.93%	−0.13 [−0.71, 0.45]
Jung et al., 2018	20	24.50	9.25	23.20	8.29	13.89%	−0.15 [−0.77, 0.48]
Martínez–Fernández et al., 2021	9	0.67	0.97	0.33	0.53	6.23%	−0.41 [−1.35, 0.52]
Nijhawan et al., 2009	31	40.00	13.33	39.00	11.11	21.27%	−0.08 [−0.58, 0.42]
Sperling et al., 2018	20	46.0	8.67	45.5	11.85	13.93%	−0.05 [−0.67, 0.57]
Witjas et al., 2015	45	15	4.44	11	6.89	28.76%	−0.68 [−1.11, −0.26]
Random Effects Model						100.00%	−0.29 [−0.52, −0.05]

Test for effect: z = −2.40, p = 0.017*

Heterogeneity: Q(df = 5) = 5.16, p = 0.40, I^2= 3.04%

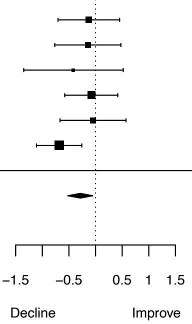

Semantic Fluency

Study	n	Baseline Mean	Baseline SD	Follow-up Mean	Follow-up SD	Weight	SMD [95% CI]
Fukuda et al., 2000	13	11.55	3.33	11.00	2.90	6.76%	−0.17 [−0.94, 0.60]
Gasca–Salas et al., 2019	23	18.13	5.38	18.78	5.41	11.99%	0.12 [−0.46, 0.70]
Jung et al., 2018	20	15.35	2.87	14.70	3.11	10.38%	−0.21 [−0.83, 0.41]
Martínez–Fernández et al., 2021	9	0.00	0.41	0.07	0.92	4.69%	0.09 [−0.83, 1.02]
Nijhawan et al., 2009	31	16.00	5.93	15.00	4.44	16.11%	−0.19 [−0.69, 0.31]
Schuurman et al., 2002	32	35.6	9.0	33.0	11.4	16.58%	−0.25 [−0.74, 0.24]
Sperling et al., 2018	20	50.0	11.85	48.0	5.78	10.39%	−0.21 [−0.83, 0.41]
Witjas et al., 2015	45	25	7.78	22	8.15	23.09%	−0.37 [−0.79, 0.04]
Random Effects Model						100.00%	−0.19 [−0.40, 0.01]

Test for effect: z = −1.91, p = 0.056

Heterogeneity: Q(df = 7) = 2.26, p = 0.94, I^2= 0.00%

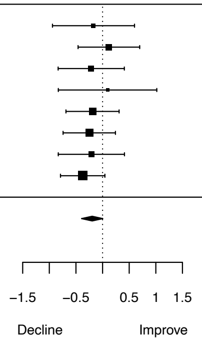

Verbal Memory: Immediate Recall

Study	n	Baseline Mean	Baseline SD	Follow−up Mean	Follow−up SD	Weight	SMD [95% CI]
Fukuda et al., 2000	13	29.18	14.00	31.27	12.98	8.86%	0.15 [−0.62, 0.92]
Gasca−Salas et al., 2019	23	7.87	1.46	7.87	1.22	15.73%	0.00 [−0.58, 0.58]
Jung et al., 2018	20	21.35	4.97	22.00	5.67	13.65%	0.12 [−0.50, 0.74]
Martínez−Fernández et al., 2021	9	7.40	2.30	8.20	0.84	6.01%	0.44 [−0.50, 1.38]
Nijhawan et al., 2009	29	−0.80	1.07	−0.52	1.24	20.35%	0.24 [−0.27, 0.75]
Schuurman et al., 2002	32	31.1	10.1	32.8	10.3	21.81%	0.16 [−0.33, 0.66]
Sperling et al., 2018	20	44.5	10.96	47.0	10.89	13.59%	0.22 [−0.40, 0.85]
Random Effects Model						100.00%	0.17 [−0.06, 0.40]

Test for effect: z = 1.46, p = 0.14
Heterogeneity: Q(df = 6) = 0.78, p = 0.99, I^2= 0.00%

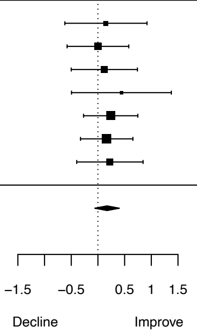

Verbal Memory: Delayed Recall

Study	n	Baseline Mean	Baseline SD	Follow−up Mean	Follow−up SD	Weight	SMD [95% CI]
Gasca−Salas et al., 2019	23	6.30	2.55	5.91	2.23	17.27%	−0.16 [−0.74, 0.42]
Jung et al., 2018	20	6.35	2.76	6.15	3.05	15.05%	−0.07 [−0.69, 0.55]
Martínez−Fernández et al., 2021	9	5.20	3.03	6.20	1.92	6.66%	0.38 [−0.56, 1.31]
Nijhawan et al., 2009	29	−0.68	0.85	−0.69	0.73	22.57%	−0.01 [−0.52, 0.49]
Schuurman et al., 2002	32	6.1	2.5	6.3	3.5	24.09%	0.06 [−0.43, 0.56]
Sperling et al., 2018	20	43.0	7.78	48.5	9.41	14.36%	0.62 [−0.01, 1.26]
Random Effects Model						100.00%	0.09 [−0.15, 0.33]

Test for effect: z = 0.73, p = 0.47
Heterogeneity: Q(df = 5) = 4.21, p = 0.52, I^2= 0.00%

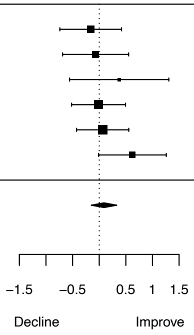

Non−Verbal Memory: Immediate Recall

Study	n	Baseline Mean	Baseline SD	Follow−up Mean	Follow−up SD	Weight	SMD [95% CI]
Jung et al., 2018	20	19.30	6.18	22.05	7.78	19.69%	0.38 [−0.24, 1.01]
Nijhawan et al., 2009	26	8.00	4.63	9.00	3.89	28.18%	0.23 [−0.29, 0.75]
Schuurman et al., 2002	32	52.1	40.3	51.5	36.7	32.09%	−0.02 [−0.51, 0.47]
Sperling et al., 2018	20	42.5	14.07	41.5	12.37	20.04%	−0.07 [−0.69, 0.55]
Random Effects Model						100.00%	0.12 [−0.16, 0.40]

Test for effect: z = 0.85, p = 0.40
Heterogeneity: Q(df = 3) = 1.52, p = 0.68, I^2= 0.00%

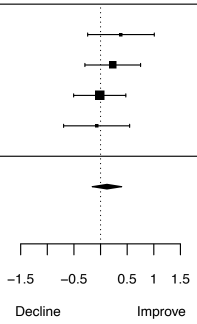

Non−Verbal Memory: Delayed Recall

Study	n	Baseline Mean	Baseline SD	Follow−up Mean	Follow−up SD	Weight	SMD [95% CI]
Jung et al., 2018	20	18.85	5.31	21.13	7.25	27.49%	0.35 [−0.27, 0.98]
Schuurman et al., 2002	32	51.5	37.6	52.2	34.4	44.67%	0.02 [−0.47, 0.51]
Sperling et al., 2018	20	44.5	12.96	46.5	13.48	27.84%	0.15 [−0.47, 0.77]
Random Effects Model						100.00%	0.15 [−0.18, 0.47]

Test for effect: z = 0.88, p = 0.38
Heterogeneity: Q(df = 2) = 0.67, p = 0.71, I^2= 0.00%

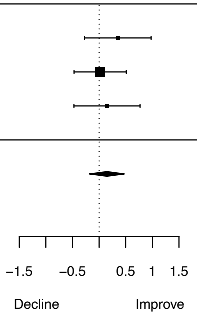

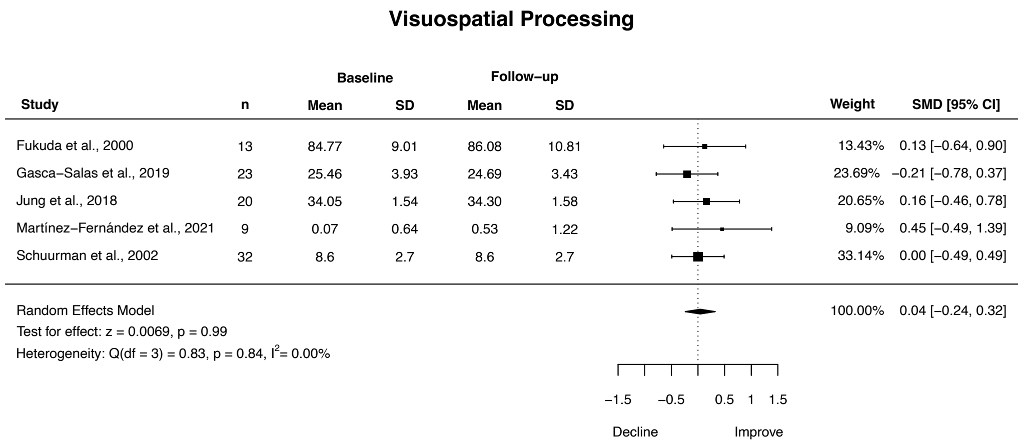

Forest plots depicting the results of the meta-analysis for all seven cognitive domains. There was a small significant decline in phonemic fluency and a trend toward a decline in semantic fluency. No significant postoperative changes were observed in domains of global cognition, executive function, verbal and non-verbal memory, and visuospatial processing. Abbreviations: n = sample size, SD = standard deviation, SMD = standardized mean difference; CI = confidence interval. * = p < .05.

Supplementary Fig. 2: Forest plots depicting the effect of magnetic resonance-guided focused ultrasound thalamotomy on cognition

Executive Functioning (Inhibition)

Study	n	Baseline Mean	Baseline SD	Follow-up Mean	Follow-up SD	Weight	SMD [95% CI]
Gasca–Salas et al., 2019	23	−67.08	15.71	−67.15	17.23	32.14%	−0.00 [−0.58, 0.57]
Jung et al., 2018	20	83.80	21.69	85.00	24.48	27.94%	0.05 [−0.57, 0.67]
Martínez–Fernández et al., 2021	9	−0.07	0.76	−0.33	0.91	12.44%	−0.30 [−1.22, 0.63]
Sperling et al., 2018	20	46.0	8.00	48.5	4.81	27.48%	0.37 [−0.25, 1.00]
Random Effects Model						100.00%	0.08 [−0.25, 0.41]

Test for effect: z = 0.47, p = 0.64

Heterogeneity: Q(df = 3) = 1.55, p = 0.67, I^2= 0.00%

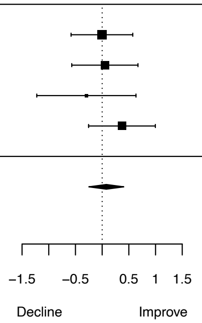

Visuospatial Processing

Study	n	Baseline Mean	Baseline SD	Follow-up Mean	Follow-up SD	Weight	SMD [95% CI]
Gasca–Salas et al., 2019	23	25.46	3.93	24.69	3.43	44.34%	−0.21 [−0.78, 0.37]
Jung et al., 2018	20	34.05	1.54	34.30	1.58	38.64%	0.16 [−0.46, 0.78]
Martínez–Fernández et al., 2021	9	0.07	0.64	0.53	1.22	17.01%	0.45 [−0.49, 1.39]
Random Effects Model						100.00%	0.05 [−0.34, 0.43]

Test for effect: z = 0.24, p = 0.81

Heterogeneity: Q(df = 2) = 1.56, p = 0.46, I^2= 0.00%

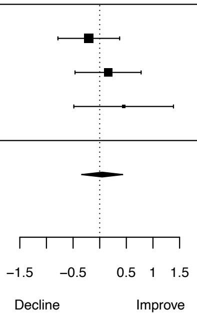

Phonemic Fluency

Study	n	Baseline Mean	Baseline SD	Follow-up Mean	Follow-up SD	Weight	SMD [95% CI]
Gasca–Salas et al., 2019	23	13.82	5.76	13.13	4.92	32.01%	−0.13 [−0.71, 0.45]
Jung et al., 2018	20	24.50	9.25	23.20	8.29	27.82%	−0.15 [−0.77, 0.48]
Martínez–Fernández et al., 2021	9	0.67	0.97	0.33	0.53	12.29%	−0.41 [−1.35, 0.52]
Sperling et al., 2018	20	46.0	8.67	45.5	11.85	27.88%	−0.05 [−0.67, 0.57]
Random Effects Model						100.00%	−0.14 [−0.47, 0.18]

Test for effect: z = −0.87, p = 0.39

Heterogeneity: Q(df = 3) = 0.42, p = 0.94, I^2= 0.00%

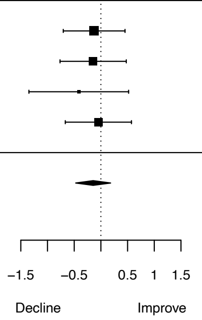

Semantic Fluency

Study	n	Baseline Mean	Baseline SD	Follow-up Mean	Follow-up SD	Weight	SMD [95% CI]
Gasca–Salas et al., 2019	23	18.13	5.38	18.78	5.41	32.01%	0.12 [−0.46, 0.70]
Jung et al., 2018	20	15.35	2.87	14.70	3.11	27.73%	−0.21 [−0.83, 0.41]
Martínez–Fernández et al., 2021	9	0.00	0.41	0.07	0.92	12.53%	0.09 [−0.83, 1.02]
Sperling et al., 2018	20	50.0	11.85	48.0	5.78	27.73%	−0.21 [−0.83, 0.41]
Random Effects Model						100.00%	−0.07 [−0.39, 0.26]

Test for effect: z = −0.41, p = 0.69

Heterogeneity: Q(df = 3) = 0.93, p = 0.82, I^2= 0.00%

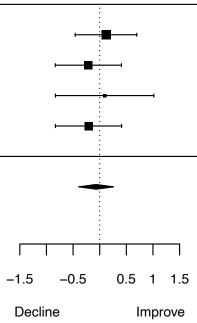

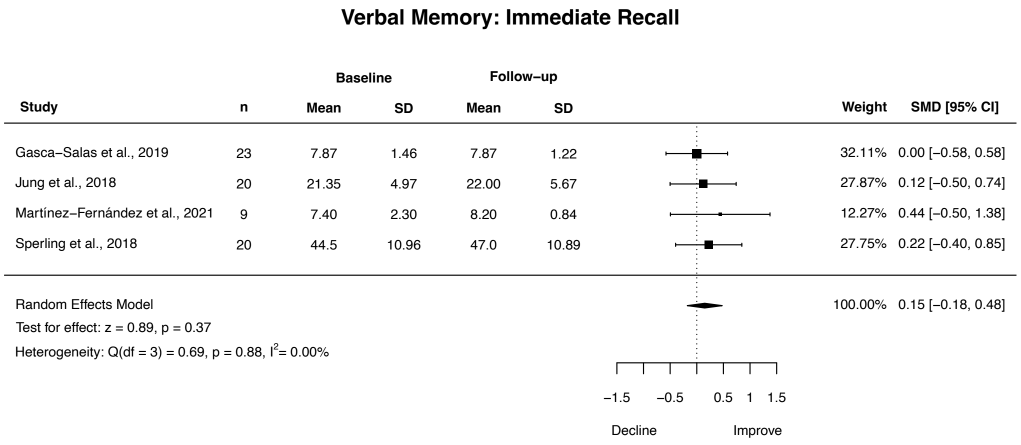

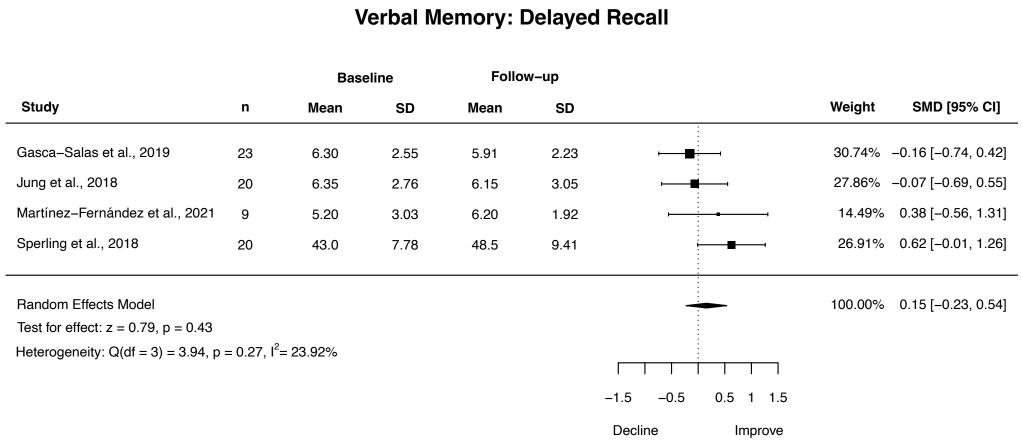

Forest plot depicting the results of the meta-analysis across cognitive domains for magnetic resonance-guided focused ultrasound. There were four studies that used magnetic resonance-guided focused ultrasound. Because we required at least three studies to perform a meta-analysis for any given cognitive domain, we only examined executive function, phonemic and semantic fluency, verbal memory, and visuospatial processing No significant postoperative changes were observed in any of these domains. Abbreviations: n = sample size, SD = standard deviation, SMD = standardized mean difference; CI = confidence interval.

Supplementary Table 2: Risk of bias

	Fukuda et al., 2000	Gasca-Salas et al., 2019	Jung et al., 2018	Martínez-Fernández et al., 2021	Nijhawan et al., 2009	Schuurman et al., 2002	Sperling et al., 2018	Witjas et al., 2015
1. Was the study question or objective clearly stated?	Yes	Yes	Yes	Yes	Yes	Yes	Yes	Yes
2. Were eligibility/selection criteria for the study population prespecified and clearly described?	No	Yes	Yes	Yes	Yes	No	Yes	Yes
3. Were the participants in the study representative of those who would be eligible for the test/service/intervention in the general or clinical population of interest?	Yes	Yes	Yes	Yes	Yes	No	Yes	Yes
4. Were all eligible participants that met the prespecified entry criteria enrolled?	Cannot determine	Yes	Yes	Yes	Yes	Yes	Yes	No
5. Was the sample size sufficiently large to provide confidence in the findings?	No	No	No	No	Yes	Yes	No	Yes
6. Was the test/service/intervention clearly described and delivered consistently across the study population?	Yes	Yes	Yes	Yes	Yes	Yes	Yes	Yes
7. Were the outcome measures prespecified, clearly defined, valid, reliable, and assessed consistently across all study participants?	No	Yes	Yes	Yes	No	Yes	Yes	Yes
8. Were the people assessing the outcomes blinded to the	Not reported	Not reported	Yes	Not reported	Not reported	Yes	Yes	Not reported

participants' exposures/interventions?								
9. Was the loss to follow-up after baseline 20% or less? Were those lost to follow-up accounted for in the analysis?	Yes	No	Yes	Yes	Yes	Yes	Yes	No
10. Did the statistical methods examine changes in outcome measures from before to after the intervention? Were statistical tests done that provided p values for the pre-to-post changes?	Yes	Yes	Yes	No	Yes	Yes	No	Yes
11. Were outcome measures of interest taken multiple times before the intervention and multiple times after the intervention (i.e., did they use an interrupted time-series design)?	NA	NA	NA	NA	NA	NA	NA	NA
12. If the intervention was conducted at a group level (e.g., a whole hospital, a community, etc.) did the statistical analysis take into account the use of individual-level data to determine effects at the group level?	NA	NA	NA	NA	NA	NA	NA	NA
Overall Rating	Fair	Fair	Good	Good	Good	Good	Good	Fair

Risk of bias was assessed using the National Institute of Health (NIH) quality assessment tool for before-after (pre-post) studies with no control group. Each of the 12 questions was assigned one of the following response options: ‘yes’, ‘no’, ‘cannot determine’, ‘not applicable’, or ‘not reported’. Studies were assigned an overall rating of good, fair, or poor. Two authors performed the assessments independently, and any disagreements were discussed and resolved.

Supplementary Table 3: GRADEpro quality of evidence assessment

Outcome measure	No of participants (studies)	Risk of bias	Inconsistency	Indirectness	Imprecision	Other considerations	Absolute (95% CI)	Certainty of the evidence	Importance
Global Cognition	98 (4 pre-post studies[a])	not serious	not serious	not serious	serious[b]	all plausible residual confounding would reduce the demonstrated effect[c]	SMD 0.1 SD lower (0.38 lower to 0.18 higher)	⊕⊕○○ Low	IMPORTANT
Executive functioning	129 (6 pre-post studies[a])	not serious	not serious	not serious	serious[b]	all plausible residual confounding would reduce the demonstrated effect[c]	SMD 0.09 SD lower (0.33 lower to 0.15 higher)	⊕⊕○○ Low	IMPORTANT
Phonemic fluency	148 (6 pre-post studies[a])	not serious	not serious	not serious	serious[b]	all plausible residual confounding would reduce the demonstrated effect[c]	SMD 0.29 SD lower (0.52 lower to 0.05 lower)	⊕⊕○○ Low	IMPORTANT
Semantic fluency	193 (8 pre-post studies[a])	not serious	not serious	not serious	serious[b]	all plausible residual confounding would reduce the demonstrated effect[c]	SMD 0.17 SD higher (0.4 lower to 0.01 lower)	⊕⊕○○ Low	IMPORTANT
Verbal memory (immediate recall)	146 (7 pre-post studies[a])	not serious	not serious	not serious	serious[b]	all plausible residual confounding would reduce the demonstrated effect[c]	SMD 0.17 SD higher (0.06 lower to 0.4 higher)	⊕⊕○○ Low	IMPORTANT
Verbal memory (delayed recall)	133 (6 pre-post studies[a])	not serious	not serious	not serious	serious[b]	all plausible residual confounding would reduce the demonstrated effect[c]	SMD 0.09 SD higher (0.15 lower to 0.33 higher)	⊕⊕○○ Low	IMPORTANT
Non-verbal memory (immediate recall)	98 (4 pre-post studies[a])	not serious	not serious	not serious	serious[b]	all plausible residual confounding would reduce the demonstrated effect[c]	SMD 0.12 SD higher (0.15 lower to 0.4 higher)	⊕⊕○○ Low	IMPORTANT
Non-verbal memory (delayed recall)	72 (3 pre-post studies[a])	not serious	not serious	not serious	serious[b]	all plausible residual confounding would reduce the demonstrated effect[c]	SMD 0.15 SD higher (0.18 lower to 0.47 higher)	⊕⊕○○ Low	IMPORTANT

Outcome measure	No of participants (studies)	Risk of bias	Inconsistency	Indirectness	Imprecision	Other considerations	Absolute (95% CI)	Certainty of the evidence	Importance
Visuospatial processing	88 (5 pre-post studies[a])	not serious	not serious	not serious	serious[b]	all plausible residual confounding would reduce the demonstrated effect[c]	SMD 0 SD (0.29 lower to 0.3 higher)	⊕⊕○○ Low	IMPORTANT

Abbreviations. CI: confidence interval, SMD: standardized mean difference, SD: standard deviation.

[a] While the included studies were pre-post designs, they were labelled as observational, before-after studies on GRADEpro, since the software had only two options for study design: randomised trial or observational study.
[b] The meta-analysis included studies with small sample sizes, and therefore certainty was downgraded based on imprecision.
[c] Practice effects in the neuropsychological tests is a confounding variable that would reduce the observed effect, thus upgrading the certainty of the evidence.

GradePro defines low certainty of evidence as having limited confidence in the effect estimate.
